# Diagnosis patterns of sickle cell disease in Ghana: a secondary analysis

**DOI:** 10.1186/s12889-021-11794-6

**Published:** 2021-09-22

**Authors:** Alexandra M. Sims, Kwaku Osei Bonsu, Rebekah Urbonya, Fatimah Farooq, Fitz Tavernier, Marianna Yamamoto, Sheri VanOmen, Brittne Halford, Polina Gorodinsky, Rachel Issaka, Tulana Kpadenou, Rhonda Douglas, Samuel Wilson, Clementine Fu, Danielle Canter, Duña Martin, Austin Novarra, Lewis Graham, Fredericka Sey, Charles Antwi-Boasiako, Catherine Segbefia, Onike Rodrigues, Andrew Campbell

**Affiliations:** 1 Children’s National Hospital, Washington, DC USA; 2 Department of Pediatrics, The George Washington University School of Medicine and Health Sciences, Washington, DC USA; 3 Cincinnati Children’s Hospital Medical Center, 3333 Burnet Ave MLC 7035, Cincinnati, OH 45229 USA; 4 University of Cincinnati College of Medicine, Cincinnati, OH USA; 5 University of Michigan Medical School, Ann Arbor, MI USA; 6 Department of General Surgery, William Beaumont Hospital, Royal Oak, MI USA; 7 Ghana Institute of Clinical Genetics, Korle Bu Teaching Hospital, Accra, Ghana; 8 Department of Physiology, University of Ghana Medical School, Accra, Ghana; 9 Department of Child Health, University of Ghana Medical School, Accra, Ghana; 10 Department of Child Health, Korle Bu Teaching Hospital, Accra, Ghana

## Abstract

**Background:**

Despite having the highest prevalence of sickle cell disease (SCD) in the world, no country in Sub-Saharan Africa has a universal screening program for the disease. We sought to capture the diagnosis patterns of SCD (age at SCD diagnosis, method of SCD diagnosis, and age of first pain crisis) in Accra, Ghana.

**Methods:**

We administered an in-person, voluntary survey to parents of offspring with SCD between 2009 and 2013 in Accra as a part of a larger study and conducted a secondary data analysis to determine diagnosis patterns. This was conducted at a single site: a large academic medical center in the region. Univariate analyses were performed on diagnosis patterns; bivariate analyses were conducted to determine whether patterns differed by participant’s age (children: those < 18 years old whose parents completed a survey about them, compared to adults: those > = 18 years old whose parents completed a survey about them), or their disease severity based on SCD genotype. Pearson’s chi-squared were calculated.

**Results:**

Data was collected on 354 unique participants from parents. Few were diagnosed via SCD testing in the newborn period. Only 44% were diagnosed with SCD by age four; 46% had experienced a pain crisis by the same age. Most (66%) were diagnosed during pain crisis, either in acute (49%) or primary care (17%) settings. Children were diagnosed with SCD at an earlier age (74% by four years old); among the adults, parents reflected that 30% were diagnosed by four years old (*p* < 0.001). Half with severe forms of SCD were diagnosed by age four, compared to 31% with mild forms of the disease (*p* = 0.009).

**Conclusions:**

The lack of a robust newborn screening program for SCD in Accra, Ghana, leaves children at risk for disease complications and death. People in our sample were diagnosed with SCD in the acute care setting, and in their toddler or school-age years or thereafter, meaning they are likely being excluded from important preventive care. Understanding current SCD diagnosis patterns in the region can inform efforts to improve the timeliness of SCD diagnosis, and improve the mortality and morbidity caused by the disease in this high prevalence population.

## Background

Sickle cell disease (SCD) is a group of genetic blood disorders, causing complications that include severe anemia, acute and chronic pain, acute chest syndrome and stroke [1, 2]. The highest prevalence of SCD is in sub-Saharan Africa, where 50–90% of children with the most common type of SCD die before their fifth birthday, often undiagnosed [2–5].

Identification of SCD in the newborn period reduces childhood death, and improves connection to prophylactic, life-saving care in a variety of resource settings [6–10]. However, no country in sub-Saharan Africa has a universal newborn screening program for hemoglobinopathies, due to financial and logistical barriers, as well as the burden of co-located diseases, such as HIV and malaria [11]. In Ghana, where 2% of newborns are estimated to have SCD [12], there has been progress in testing newborns, but only through piloted, local programs [13]. Targeted newborn screening for SCD in Ghana started in the mid-1990s, and more than 170,000 newborns have been screened to date, with most maintaining necessary follow-up [14, 15]. However, the lack of a sustained national approach in Ghana may result in underdiagnosis of SCD, underutilization of life-saving strategies (such as prophylactic penicillin, vaccinations, or anticipatory guidance to parents), and the disease may not be identified until pain crisis, infection or even death present.

Early diagnosis of SCD is critical. Given the lack of universal newborn testing for SCD in Ghana, we sought to better understand the existing diagnostic patterns for SCD in Accra, and to determine whether patterns differ by age or disease severity based on SCD genotype. We queried parents to learn how their child’s SCD was first diagnosed. We hypothesized that SCD would be diagnosed in the acute setting, that few would be diagnosed in infancy, and that those with more severe disease would be diagnosed earlier in life.

## Methods

This study is a secondary data analysis of the “Determining the Sickle Cell Phenotype within the Ghanaian SCD population: A Cross-sectional Analysis of Pediatric and Adult Sickle Cell Patients” study in Accra, Ghana at Korle Bu Teaching Hospital (KBTH). Accra is the capital of Ghana and is on the country’s southern coast. KBTH is academically affiliated with the University of Ghana, is the third largest hospital in Africa, and is the only public tertiary care center in Ghana’s southern region. It serves as a major referral center in the area.

We analyzed single site data from KBTH collected between 2009 and 2015. Parents were eligible for participation if they had a child (of any age) with SCD with laboratory confirmation who attended the KBTH sickle cell clinic. Parents completed a voluntary, in-person questionnaire about their child’s SCD, available in English and translated into their native dialect (Ga, Twi, etc.), if needed. Parents were asked “how were you informed that your child had sickle cell disease?” with the following response options: *blood test after presenting to hospital/emergency room with pain crisis; blood test after presenting to pediatrician’s office with pain crisis; blood test performed because another child in family had sickle cell disease; blood test performed at birth (newborn screen); blood test performed after presenting to pediatrician’s office/hospital/ER with illness (not pain crises); other*; and *unknown*. Though this is not reflective of all clinical scenarios in which SCD could present for the first time, pain crisis is quite common in SCD, and is the most noticeable complication that parents can discern with relative ease. We captured the mechanism of new SCD diagnosis and child’s age at the time of diagnosis. This study was approved by the Institutional Review Board (IRB) at the University of Michigan Medical School and the Noguchi Memorial Institute for Medical Research IRB at the University of Ghana. This sub-analysis was approved as exempt by the IRB at Children’s National Hospital.

For the purposes of analysis and clear reporting, the “child” who’s SCD presentation and diagnosis the parent was reporting was considered the participant and was categorized as either a “child” (< 18 years old) or an “adult” (> = 18 years old) based on their age at the time of the parent’s completion of the survey. This terminology will be used henceforth. Univariate analyses were performed on diagnostic modality for the participant’s SCD; bivariate analyses determined whether diagnosis patterns differed by the participant’s age group or disease severity. Severe disease was defined as hemoglobin (Hb)-SS or Hb S-beta thalassemia-zero; mild disease was defined as Hb SC, Hb S-beta thalassemia-plus disease, and other rare forms of SCD [16]. Pearson’s chi-squared was used to compare the frequencies between groups. The level of statistical significance was set at *p* < 0.05. Fisher’s exact was used in cases where cells were present with values less than five. Quantitative analysis was conducted using STATA IC Version 15 [17]*.*

## Results

Data from a total of 354 unique participants were collected via questionnaires completed by their parents. There were minimal missing data, which is reflected in Tables 1, 2 and 3. The median age for participants was 23 years (Table 1). Sixty-eight percent of the participants were adults (> = 18 years old). Half (54%) were female. Nearly all were of Ghanaian descent. Two-thirds had Hb-SS disease.

**Table 1:** Participant Demographics. As reported via survey by parent

Variable	All (*n* = 354)
**Age** (years)	
Median (IQR)	23 (13–32)
**Adults** (> = 18 years)	235 (67.7%)
**Children** (< 18 years)	112 (32.3%)
*Missing age = 7*	
**Sex**	
Male	164 (46.5%)
Female	189 (53.5%)
*Missing sex = 1*	
**Patient Genotype**	
**Severe SCD**	
Hb SS	230 (65.5%)
Hb S Beta Thalassemia-zero	5 (1.4%)
**Mild SCD**	
Hb SC	111 (31.6%)
Hb S Beta Thalassemia-plus	3 (0.9%)
Other	2 (0.6%)
*Missing genotype = 3*	
**Age of SCD Diagnosis**	
0–6 months old	39 (11.0%)
7–11 months old	19 (5.4%)
1–2 years old	56 (15.8%)
3–4 years old	43 (12.2%)
5–10 years old	101 (28.5%)
11–14 years old	41 (11.6%)
15+ years old	55 (15.5%)
*No missing data*	
**Mechanism of SCD Diagnosis**	
“Blood test…”	
For pain crisis	
At the hospital/ER	169 (49.3%)
At the pediatrician’s office	57 (16.6%)
Because child in family had SCD	26 (7.6%)
At birth (newborn screen)	19 (5.5%)
For another illness (not pain crisis)	55 (16.0%)
Other	17 (5.0%)
*Missing diagnosis mechanism = 11*	

*Hb* Hemoglobin

*ER* Emergency room

*SCD* Sickle cell disease

**Table 2:** Participant Diagnosis Patterns, by Age Group. As reported via survey by parent

Variable	Adults (> = 18 years) (*n* = 235)	Children (< 18 years) (*n* = 112)
**Age of SCD Diagnosis (*p*** **< 0.001)**		
0–6 months old	27 (11.1%)	12 (10.7%)
7–11 months old	4 (1.7%)	15 (13.4%)
1–2 years old	20 (8.5%)	34 (30.4%)
3–4 years old	20 (8.5%)	22 (19.6%)
5–10 years old	78 (33.2%)	22 (19.6%)
11–14 years old	34 (14.5%)	6 (5.4%)
15+ years old	53 (22.6%)	1 (0.9%)
*No missing data*		
**Mechanism of SCD Diagnosis (*p*** **< 0.001)**		
“Blood test…”		
For pain crisis		
At the hospital/ER	116 (51.3%)	48 (43.2%)
At the pediatrician’s office	43 (19%)	13 (11.7%)
Because child in family had SCD	5 (2.2%)	21 (18.9%)
At birth (newborn screen)	18 (8.0%)	1 (0.9%)
For another illness (not pain crisis)	32 (14.2%)	23 (20.7%)
Other	12 (5.3%)	5 (4.5%)
*Missing diagnosis mechanism = 11*		
**Age of First Pain Crisis (*p*** **< 0.001)**		
0–6 months old	16 (8.7%)	2 (2.0%)
7–11 months old	1 (0.5%)	2 (2.0%)
1–2 years old	30 (16.2%)	46 (45.1%)
3–4 years old	12 (6.5%)	20 (19.6%)
5–10 years old	69 (37.3%)	28 (27.5%)
11–14 years old	21 (11.3%)	3 (3.0%)
15+ years old	36 (19.5%)	1 (1.0%)
*Missing first pain crisis = 64*		

*ER* Emergency room

*SCD* Sickle cell disease

Fisher’s exact used where individual cells < 5

**Table 3:** Participant Diagnosis Patterns, by Disease Severity (based on genotype). As reported via survey by parent

Variable	Severe SCD (*n* = 235)	Mild SCD (*n* = 116)
**Age of SCD Diagnosis (*p*** **= 0.009)**		
0–6 months old	30 (12.7%)	9 (7.8%)
7–11 months old	15 (6.3%)	4 (3.5%)
1–2 years old	44 (18.6%)	12 (10.3%)
3–4 years old	31 (13.1%)	11 (9.5%)
5–10 years old	64 (27.0%)	37 (31.9%)
11–14 years old	27 (11.4%)	14 (12.1%)
15+ years old	26 (11.0%)	29 (25.0%)
*No missing data*		
**Mechanism of SCD Diagnosis (*p*** **= 0.132)**		
“Blood test…”		
For pain crisis		
At the hospital/ER	105 (46.1%)	63 (55.3%)
At the pediatrician’s office	43 (18.9%)	14 (12.3%)
Because child in family had SCD	15 (6.6%)	11 (9.7%)
At birth (newborn screen)	15 (6.6%)	4 (3.5%)
For another illness (not pain crisis)	41 (18.0%)	14 (12.3%)
Other	9 (4.0%)	8 (7.0%)
*Missing diagnosis mechanism = 11*		
**Age of First Pain Crisis (*p*** **= 0.047)**		
0–6 months old	14 (7.3%)	4 (4.1%)
7–11 months old	3 (1.6%)	0 (0%)
1–2 years old	62 (32.3%)	17 (17.4%)
3–4 years old	18 (9.4%)	14 (14.3%)
5–10 years old	58 (30.2%)	39 (39.8%)
11–14 years old	16 (8.3%)	8 (8.2%)
15+ years old	21 (10.9%)	16 (16.3%)
*Missing first pain crisis = 64*		

*ER* Emergency room

*SCD* Sickle cell disease

Severe SCD: Hb SS; Hb S Beta Thalassemia-zero

Mild SCD: Hb SC; Hb S Beta Thalassemia-plus; Other

Fisher’s exact used where individual cells < 5

Only 5.5% were diagnosed with SCD in the newborn period (8% of adults, 1% of children). Two-thirds were diagnosed during pain crisis, either in the hospital or emergency department setting (49%) or at the pediatrician’s office (17%). Forty-four percent were diagnosed with SCD by age four, and a similar number had experienced a pain crisis by the same age (46%).

We next analyzed responses based on participant age at the time of their parent’s completion of the survey to determine whether diagnostic patterns differed in each of these groups (Table 2). Children were diagnosed with SCD earlier (74% by four years old); 30% of the adult participants were diagnosed by age four (*p* < 0.001). Children were less likely to be diagnosed during a medical encounter for pain crisis (55%) in comparison to adults (70%). Children were more likely to be tested and diagnosed because another child in the family had SCD (19%, compared to 2% of adults) (*p* < 0.001). Thirty-two percent of the adult participants presented for first pain crisis by age four years, compared to 69% of children (*p* < 0.001). Children were less likely to be screened for SCD at birth (1%, compared to 8% of adult participants; *p* < 0.001).

We explored whether SCD severity (assessed by genotype) was associated with diagnostic patterns. Two-thirds of all participants had severe SCD, and one-third had mild disease (Table 3). Half with severe SCD were diagnosed with SCD by age four, compared to 31% with mild SCD (*p* = 0.004). There were not significant differences in diagnosis method based on SCD severity (*p* = 0.132). As expected, those with severe disease experienced pain crisis earlier in life (51% by age four years) compared to those with mild disease (36% by age four years) (*p* = 0.047).

## Discussion

Our findings indicate that SCD in this population is diagnosed in acute settings, with pain as a common first presentation of the disease. Most of our participants had not been diagnosed with SCD by four years old based on parent report, reflecting missed opportunities for timely diagnosis, and likely missed preventive care, in early childhood.

These diagnosis patterns are problematic for a myriad of reasons. Individuals with SCD are unintentionally excluded from life-saving preventative care when they are not identified early. SCD increases the risk for infections caused by *Streptococcus pneumoniae* and *Haemophilus influenzae*. Prior to vaccinations, these infections were the cause for most pediatric SCD deaths [18]. After the introduction of the first pneumococcal conjugate vaccine, invasive pneumococcal disease decreased by 93% in children under 5 years old [19]. Prophylactic treatment with penicillin in children soon after SCD identification has similarly improved morbidity and mortality in pediatrics SCD [20]. However, gaps remain in Ghana. Dayie et al. recently found that under half of children with SCD had received full immunization against *S. pneumoniae* and very few were on penicillin prophylaxis; this was in a study population very similar to ours [21]. The significant morbidity and mortality related to SCD, particularly in lower-resourced countries, also places a strain on the health care system that could be partially mitigated with early identification and improved management of disease [22].

Some of our findings reflect an encouraging trend: participants < 18 years old were, for example, more likely to be diagnosed with SCD by age four compared to those > = 18 years old, had fewer SCD diagnoses in the acute setting, and more SCD diagnoses prompted by targeted testing due to a family history of SCD. However, nearly the same percentage of adults and children with SCD were diagnosed by six months old, meaning those who were born more recently do not fare better regarding SCD diagnosis during early infancy. And, screening in the newborn period was *not* identified as a major diagnosis modality, even among children born relatively recently. This is, perhaps, in contrast to successful systematic screening and management campaigns worldwide. Newborn SCD screening in our study population has been largely executed via temporary and/or targeted programs: between 1995 and 2003, over 200,000 newborns were screened in a variety of clinical settings through a demonstration project [12, 23]. In a rural, low resource setting in Ghana, nearly 400 newborns were screened more recently via point of care testing in a multisite trial [13]. But screening availability has fluctuated over time, whereby a parent might have had access to a SCD newborn screening program for a first-born child, but this program might have been unavailable for subsequently born children. Our data was limited in that we are not able to say whether the few participants who were diagnosed at birth (*n* = 19) were reached by pilot initiatives, and if so, which programs. Despite scientific advancement, historical problems persist in early SCD diagnosis in under resourced settings, particularly in infancy. Further, genetic counseling remains unavailable to most Ghanaians with SCD or for those who are carriers, despite the World Health Organization’s support of this practical and cost-effective strategy [24]. Universal newborn screening in this region, which our data supports, will facilitate connecting children to vital care early in life, relieve health care expenditures, and better distribute the health care workforce [25]. Multidisciplinary collaboration as well as community engagement have been identified as important factors in successful screening efforts [26, 27].

While awaiting a national approach, there may be clinical considerations for providers that our research helps to elevate. SCD screening that is prompted by having another child in the family with SCD is a recognized method of diagnosis in our study population, more common in those born more recently (8% overall; 19% of those < 18 years old, compared to only 2% of those > = 18 years old). Providers in resource limited settings may consider using careful family history as a part of their practice for identifying SCD risk.

Our survey response options for diagnosis method largely focused on pain-related clinical presentations. Pain is not the only presentation of SCD crisis but is a common one – recent work with adults with SCD at KBTH demonstrated that pain was the most frequently reported symptom [28]. Pain is also recognizable by parents with observation alone, and without reliance on technologies, such as a thermometer for identifying fever. Our results support this framing – only 16% presented with an illness other than pain crisis at the time of their diagnosis.

There are certainly limitations in this work. First, selection bias is present. Those that live in remote or rural locations may not be reflected in the results, given that our site was an academic medical center in a large city; in fact, this underrepresented group likely faces greater health care access issues. Second, parents in this study were asked to reflect on the health of their children retrospectively. Given the wide age range of the participants (some still in childhood and others who had reached adulthood by the time of their parent’s completion of the survey), there is likely significant recall bias present. We also did not control for other factors that may prime parents to seek out SCD screening, such as birth order, whether the family had other children with SCD or history of child death in the family.

In conclusion, this work affirms that, by parent report, SCD is diagnosed in Ghana during first pain crisis in toddler and school-age years. This unfortunately means that many are being excluded from life-saving preventive care. These strategies – penicillin prophylaxis, immunization, and hydroxycarbamide – *are* available in Accra, and industries and government are collaborating to address affordability and access [29]. However, they will be completely out of reach if SCD is unidentified. Relying on SCD diagnosis during first pain crisis or targeted childhood screening based on having a sibling with SCD is suboptimal; even children diagnosed with SCD during early childhood miss out on prevention strategies that would otherwise be implemented if SCD was identified soon after birth. A recent multidisciplinary international partnership with KBTH has demonstrated feasibility, acceptability and need for SCD screening in the newborn period [30]. There remain opportunities for early SCD identification as a part of a comprehensive, universal approach, starting with diagnosis and complication prevention in the newborn period, and stretching across the lifespan to prevent untimely death [31, 32].

## Data Availability

The datasets used and/or analyzed during the current study are available from the corresponding author on reasonable request.
